# Editorial: Metabolome Informatics and Statistics: Current State and Emerging Trends

**DOI:** 10.3389/fbioe.2016.00063

**Published:** 2016-07-19

**Authors:** Adam James Carroll, Reza M. Salek, Masanori Arita, Joachim Kopka, Dirk Walther

**Affiliations:** ^1^Research School of Biology, Australian National University, Canberra, ACT, Australia; ^2^European Molecular Biology Laboratory, European Bioinformatics Institute (EMBL-EBI), Wellcome Trust Genome Campus, Cambridge, UK; ^3^National Institute of Genetics, Mishima, Japan; ^4^Max Planck Institute of Molecular Plant Physiology, Potsdam-Golm, Germany

**Keywords:** metabolomics/metabolite profiling, mass spectrometry, nuclear magnetic resonance, secondary metabolism, primary metabolism, data interpretation, statistical, databases as topic

**The Editorial on the Research Topic**

**Metabolome Informatics and Statistics: Current State and Emerging Trends**

Metabolomics has developed tremendously since the term “metabolome” was coined almost 20 years ago (Oliver et al., 1998). Once the domain of few laboratories, it is now a core capability at most major universities and research institutions. An important developmental indicator for any scientific discipline is the maturity of its informatics – the functional diversity and efficacy of computational and statistical approaches, software tools, databases, and data exchange standards that help transform raw data into understanding. The 13 articles from 81 authors in this frontiers research topic provide a snapshot of the current state of these platforms.

Metabolomics offers unique challenges for software developers. One of the most fundamental is the development of databases enabling the structures and properties of metabolites to be queried in ways that enhance research. In this research topic, Johnson and Lange review the development of open-access spectral reference databases to aid natural product identification. Maeda describes 3DMET^1^ – a database of metabolite three-dimensional (3D) structures – using software to convert 2D representations of 3D structures from printed articles to 3D digital structure models.

The diversity of analytical techniques used in metabolomics is wider than in other omics disciplines, and the list of relevant technologies continues to grow. Thus, each technique is typically associated with a swathe of literature describing specialized computational methods and software tools, and there is always demand for new software to support new methods. The review article “Analytical methods in untargeted metabolomics: state of the art in 2015” provides a useful overview of this area (Alonso et al.).

For the processing of liquid chromatography mass spectrometry (LC-MS) data, Tsugawa et al. describe multiple reaction monitoring (MRM)-DIFF – a powerful pipeline for MRM-based analysis of lipidomic samples on LC-triple quadrupole MS instruments. As a demonstration of novel approaches for LC-MS peak identification, van der Hooft et al. describe how an analysis of high resolution MS^n^ fragmentation spectra using freely available MAGMa software^2^ could be used to annotate peaks of 50 different acylcarnitines in human urine.

For gas chromatography-MS (GC-MS) metabolomics data processing, Kuich et al. introduce Maui-VIA – a GUI-based tool that streamlines the visual curation of peak identifications and quantifications. Also for GC-MS, Franceschi et al. describe MetaDB, a web application providing an user-friendly web interface and laboratory information system (LIMS)-like handling of workflow metadata to metaMS – their R-based data processing pipeline for untargeted quantitative GC/MS metabolomics. Trutschel et al. demonstrate that the joint analysis of multiple dependent signals from the same metabolite (e.g., multiple fragments) using multivariate statistical tests can provide enhanced statistical power to detect differential metabolite abundance than the typical univariate analysis of single signals.

Another exciting area of metabolome informatics and statistics is the development of computational approaches to assist biological interpretation. Sun et al. discuss the potential to derive quantitative information about causality networks responsible for metabolome dynamics from metabolomics data and metabolic models by inverse Jacobian estimation. Kessler et al. demonstrate machine learning-based classification of crops as having been “organically” or “non-organically” grown. Uppal et al. present MetabNet – an R package to detect associations between metabolites of interest and peaks detected in LC-MS experiments for the purposes of detecting likely metabolic pathway connections. Finally, Carroll et al. (2010) presented PhenoMeter – a tool for functionally annotating query metabolic phenotypes by matching them against the MetabolomeExpress phenotypic reference database just as BLAST searches are used to annotate nucleotide or protein sequences (Carroll et al.).

With so many metabolomics datasets in the literature and the rate of data generation increasing, the need to systematically index and annotate them has never been more urgent. To this end, Metabolonote^3^ of Ara et al. takes the innovative approach of using a Wiki style interface to facilitate community-based metadata annotation of metabolomics datasets from the literature, delocalizing the burden of this work. The metadata handling functions of the MetaDB pipeline described by Franceschi et al. also aim to streamline the annotation of datasets while supporting the standard ISA-Tab format for dissemination through the MetaboLights^4^ data repository (Haug et al., 2012).

Metabolome informatics and statistics is incredibly broad and fast moving, and this research topic can therefore offer only a cross-sectional sample of developments at one point in time. The cutting edge developments of the future will be built upon those of the present and in a field as rapidly evolving as metabolomics, it is particularly critical and challenging to ensure one’s own work builds upon and benefits from the efforts of others as much as possible. We hope the reader finds this research topic a useful contemporary reference to the field that informs and inspires exciting future innovations and collaborations that help realize the full potential of metabolomics.

## Author Contributions

This manuscript was written by AC. It was revised and edited by RS, MA, JK, and DW prior to submission.

## Conflict of Interest Statement

The authors declare that the research was conducted in the absence of any commercial or financial relationships that could be construed as a potential conflict of interest.

## Footnotes

^1^http://www.3dmet.dna.affrc.go.jp/

^2^http://www.emetabolomics.org/

^3^http://metabolonote.kazusa.or.jp/

^4^http://www.ebi.ac.uk/metabolights/
